# The earliest Baltic amber in Western Europe

**DOI:** 10.1038/s41598-023-41293-0

**Published:** 2023-08-31

**Authors:** M. Murillo-Barroso, A. Martín Cólliga, M. Martinón-Torres

**Affiliations:** 1 Departamento de Prehistoria y Arqueología, Universidad de Granada, Campus de Cartuja s/n, 18071 Granada, Spain; 2 Government Institution of Catalonia-Generalitat de Catalunya, Archaeological and Paleontological Service, Catalonia, Spain; 3 Department of Archaeology, University of Cambridge, Downing Street, Cambridge, CB2 3ER UK

**Keywords:** Socioeconomic scenarios, Characterization and analytical techniques

## Abstract

The occurrence of Baltic amber through Europe has traditionally been associated to the spread of the Bell Beaker culture during the 3rd millennium BC. In Iberia, this phenomenon is particularly noticeable in the southern half. Here we present an amber bead recovered in a Late Neolithic funerary cave (3634–3363 cal BC) from northeastern Iberia where more than 12 individuals had been buried. Fourier transform infrared spectroscopy results of four samples revealed their complete resemblance with Baltic succinite reference spectra. Despite being a single bead, this finding provides the earliest evidence for the arrival of Baltic amber to the Mediterranean and Western Europe, before the Bell Beaker phenomenon and more than a millennium earlier than traditionally thought. This finding has implications for our understanding of early exchange networks of exotic materials, and their associated social structures.

## Introduction

Objects made from 'exotic' raw materials are key elements of archaeological material culture. From a production perspective, they can inform about trade and exchange, mobility and craft organisation; their consumption patterns are often connected to issues of social status, identity and gender.

Extended cultural networks were as fundamental in the past as they are in the present. Specifically, long-distance trade networks could enable privileged access to knowledge, technologies, objects and social relations. Similarly, the restricted availability of certain materials could have generated prestige and other forms of social differentiation. On the one hand, networks can facilitate association and mutual assistance in times of threat, with the social relationships established through exchange being more important than the exchanged objects themselves^1^. But cooperative survival networks also trigger dependence, social debt and competition, potentially leading to social imbalances^2^. It is therefore important to consider how long-distance trade and exotica served as symbolic resources. What impact did the former have on the movement of objects, technologies and ideologies that enabled certain people (individuals, groups or whole communities) to maintain and consolidate their social status, power and influence^3^? And how was long-distance trade related to other processes of aggregation, fission or factional competition in their association with social power or prestige^4,5^?

During Late Prehistory in Europe the use of scarce and unusual raw materials (the so-called “exotica”) expanded greatly. These ranged from organic resources, such as ivory, ostrich eggshell, amber or jet, to a myriad of abiotic materials, including obsidian, rock crystal, cinnabar, and the earliest metals^3,6,7^. However, not all these resources had the same social significance nor were all distributed and circulated following the same patterns. The social value they acquired depended on several interrelated factors that varied throughout their social life, spatially and temporally depending on the context^6^. We can distinguish three stages in which materials acquired different values: at the time of their manufacture (given the particular requirements of raw materials and potentially specialised knowledge or skills); during their use (both during their useful life and when it came to discarding or depositing them in ritualised contexts); and equally importantly, at the moment of their exchange, depending on their rarity and role in local, mid- or long-distance trading networks and social relationships^6^.

Between 3500 and 2200 cal BC, we observe intense interactions and trade in objects in the Western Mediterranean region, where (leaving the Atlantic façade aside), two different systems seem to have been operating^2,8^—a reflection of the importance and dynamism of the trading networks in this area. The first system spanned the southern half of Iberia, North Africa and Sicily and involved the exchange of ostrich eggshell, ivory and Sicilian amber (simetite)^9–11^. Sicilian amber is documented for the first time in southern Iberia and Sicily in the 4th millennium BC^10,12–14^ and is widely documented during the 3rd millennium BC, usually associated with ivory and sometimes ostrich eggshell in southern Iberia^9^. At this time we also find copper objects and Bell Beaker pottery in northwest Africa; they are assumed to have originated in Iberia and were supposedly exchanged for ivory or ostrich eggs^15^.

The second proposed network encompassed northeastern Iberia, southern France, Sardinia and Italy. In the northeast of the Iberian Peninsula, exchange networks with the south of France became very intensive^16–18^. The floruit arrived with the "Sepulcros de Fosa" culture, known for its necropoleis of individual tombs, many of them with high-quality grave goods. The most highly prized autochthonous precious stone was the variscite from the Can Tintorer mines in Gavà, which was used to make necklace beads, pendants and bracelets that spread beyond northeastern Iberia through trading networks^19,20^, as documented for example in French megaliths from the 4th millennium BC^16,21^. Among the allochthonous carved materials, Provençal honey-coloured flint from Vaucluse (France) reached northeastern Iberia in large quantities and, although local flint was also used, the honey-coloured stone was almost exclusively reserved for burials. The Sardinian obsidian from Lipari and Pantelleria that was circulating in the Central Mediterranean reached the northwestern Mediterranean with the Chasséen^22^ and “Sepulcros de Fosa” cultures^23,24^, between the 5th and 4th millennia BC, and North Africa, between the 6th and the 2nd millennia BC^25,26^. It only occasionally arrived to northeastern Iberia^23,24^, possibly alongside other products^16,22^. In addition to polished stone tools, such as hornfels, made of local stone, we document exogenous specimens including Alpine jade, serpentinite from the Pyrenees, cinerite from Requista del Aveyron (France), and calcium amphibolite from the Pyrenees or the Alps^16,17,22,27–29^. Turning to pottery, Chassey vessels and French decorations have been recorded in the Iberian northeast, together with square-mouthed ceramics from La Lagozza, Italy, although the latter are less abundant^30,31^. The presence of allochthonous materials of diverse origins demonstrates the intense activity of the trade networks at that time, and confirms the unequal access to all these goods^20,32–34^.

The distribution of amber in France during the Copper Age (3000–2200 cal BC) was mainly concentrated on the Mediterranean coast and the Paris Basin, with only scant finds in Brittany^35^. The few archaeological samples from this period hitherto analysed revealed a Baltic origin^36,37^, but French amber deposits may also have been exploited, as documented in earlier and later periods^37^. In the Iberian Peninsula, recent studies have allowed us to observe the fluctuations in the consumption patterns of amber both spatially and temporally^9^. The earliest evidence of amber use dates to the Upper Palaeolithic in the north of the Peninsula^38^, initiating a tradition of exploitation of local amber resources that would last until the Bronze Age; no Sicilian simetite has been documented in Northern Iberia to date^9^. In the southern half of the Peninsula, the earliest evidence of amber is documented in the Neolithic, with a strong presence of Sicilian amber, especially from the 3rd millennium BC onwards^9,10,14^. With the exception of the Iberian northeast, amber virtually disappears from the archaeological record until the Late Bronze Age/Early Iron Age, possibly related to population movements in the Mediterranean^9^. By then, all documented amber is already of Baltic origin, with amber from other sources, including local, disappearing from the Iberian archaeological record^9^.

In northeastern Iberia, the well-dated contexts with amber have so far been dated from the 2nd millennium BC onwards, although some come from collective funerary contexts with amber containing both 3rd and 2nd millennia materials^39^. Here we present the earliest evidence of the arrival of Baltic amber on the Iberian Peninsula, dated to the middle of the 4th millennium BC. The artefacts were found in Cova del Frare (Matadepera, Barcelona) and probably arrived via the “Sepulcros de Fosa” culture trade networks, before their proposed collapse^34,40^.

## The archaeological context: Cova del Frare (Matadepera, Barcelona)

**Figure 1:**
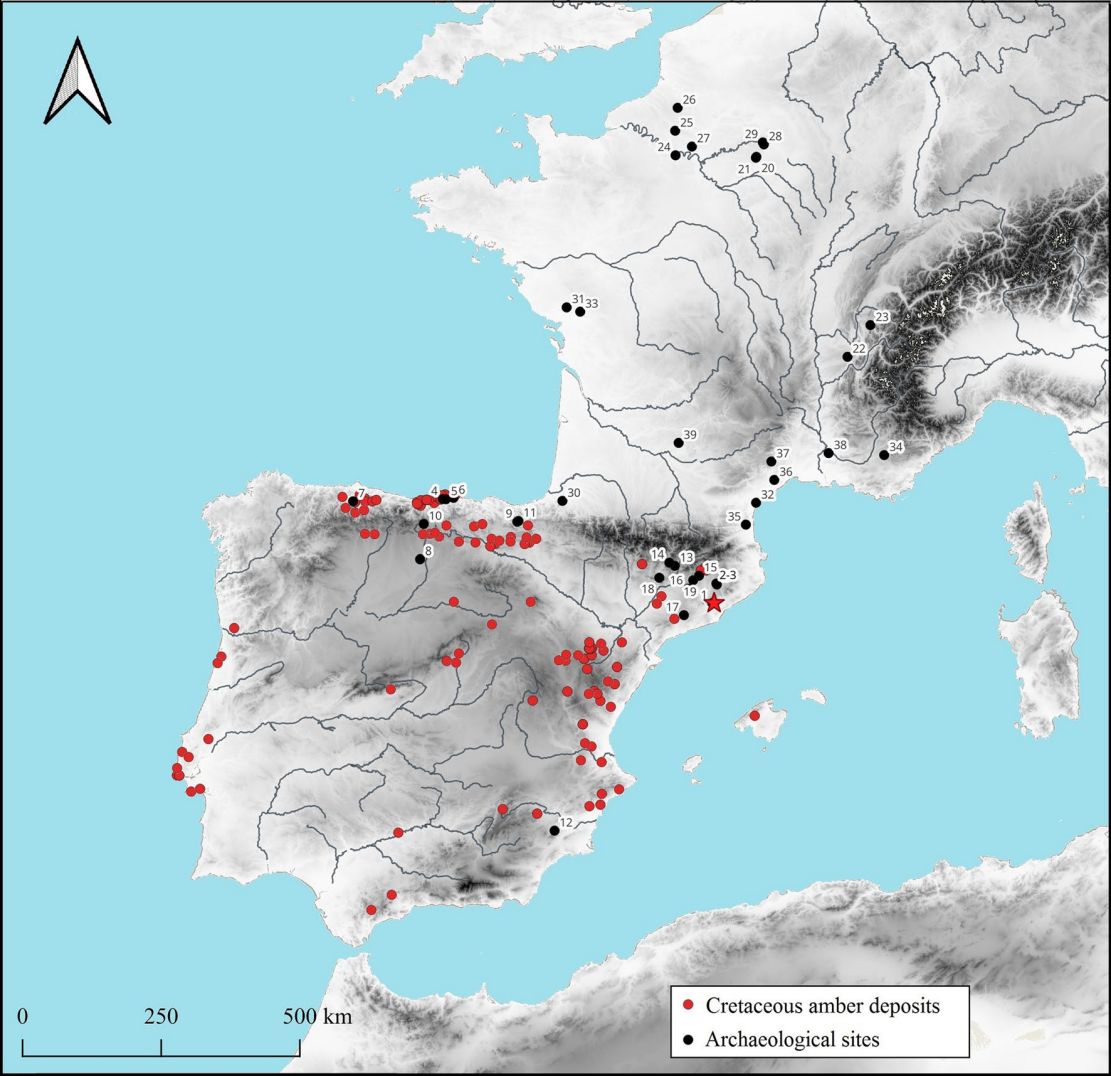
Location of Cova del Frare (red star) and all sites mentioned in the text. 1. Cova del Frare, 2. Bòbila Madurell, 3. Can Gambús, 4. El Pendo, 5. Morín, 6. La Garma A, 7. Las Caldas, 8. La Velilla, 9. Trikuaizti I, 10. Los Lagos I, 11. Larrarte, 12. La Almoloya, 13. Cabana del Moro de Colomera, 14. Pedra Cabana, 15. El Bosc, 16. La Pera, 17. Cova de El Garrofet, 18. Muricecs, 19. Fossa del Gegant, 20. Villevenard, 21. Oyes, 22. Charavines, 23. Annecy, 24. Epone, 25. Flavacourt, 26. Méréaucourt, 27. Mériel, 28. Chouilly, 29. Ay Champagne, 30. Isturitz, 31. Thiré, 32. Narbonne, 33. Xanton-Chassenon, 34. Montagnac-Montpezat, 35. Salses, 36. Saint-Pargoire, 37. Saint Maurice-de-Navacelles, 38. Châteaurenard, 39. Montpezat. Amber deposits in Iberia are also indicated.

Cova del Frare (Matadepera, Barcelona) is 960 m above sea level, near the pre-coastal mountain range which connects the fertile lands of the pre-coastal depression to the Ebro depression (Fig. 1). The cave is part of an area well connected to its surroundings and would have been a reference point since prehistoric times, judging from the stratigraphic sequence documented during the excavations undertaken between 1977 and 1984 under the supervision of one of us (A.M.C.)^41,42^. The chronology covers the Early Neolithic to the Bronze Age, as confirmed by diagnostic finds and C14 dating (see below).

**Figure 2:**
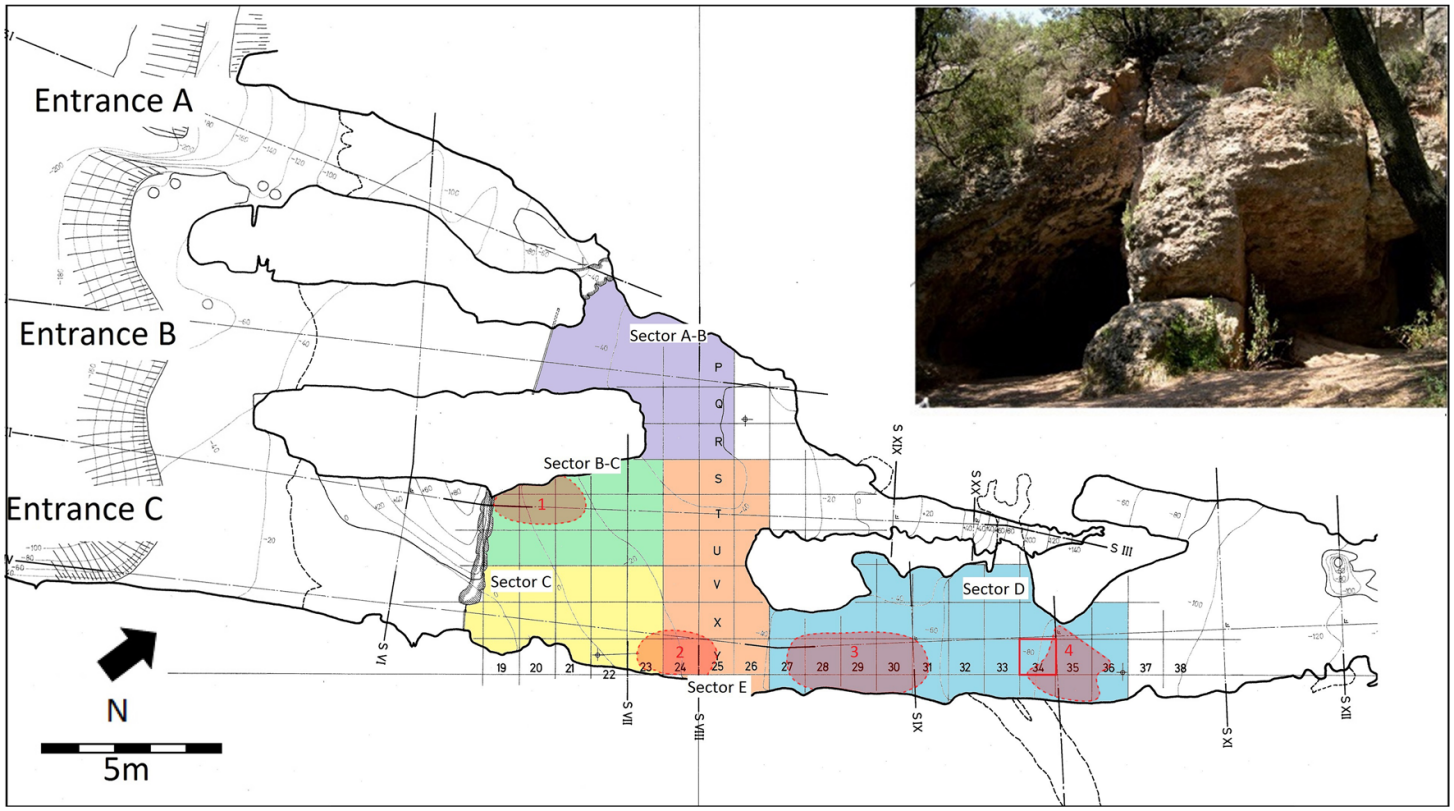
Topography of the cave. Sectors A-B, B-C, C, D and E are shown in different colours. Zones 1–4 where bones were concentrated are indicated. Square Y34, where the amber bead was found, is highlighted in red. In the picture, blocks B and C at the entrance can be observed. Adapted from Martín Cólliga et al.^44^: Fig. 1.

The cave is about 70 m long (the interior half of which is inaccessible) and has three entrances that open to the south-southwest. The finds were concentrated in the corridor and around Entrance C. The main room is spacious and well-lit by the other two entrances (A and B)^42–45^(Fig. 2).

An amber bead was found among the grave goods of those buried in the cave in the middle of the 4th millennium BC. The funerary level in question is an enclosed area that allows us to analyse the ritual and taphonomic effects without contamination from other levels, given that the lower and upper levels correspond to habitation^45^. This horizon is found below a Chalcolithic Bell Beaker level, either on sterile sediment or on remains from the Middle Post-Cardial Neolithic or Early Epicardial Neolithic, depending on the area.

**Figure 3:**
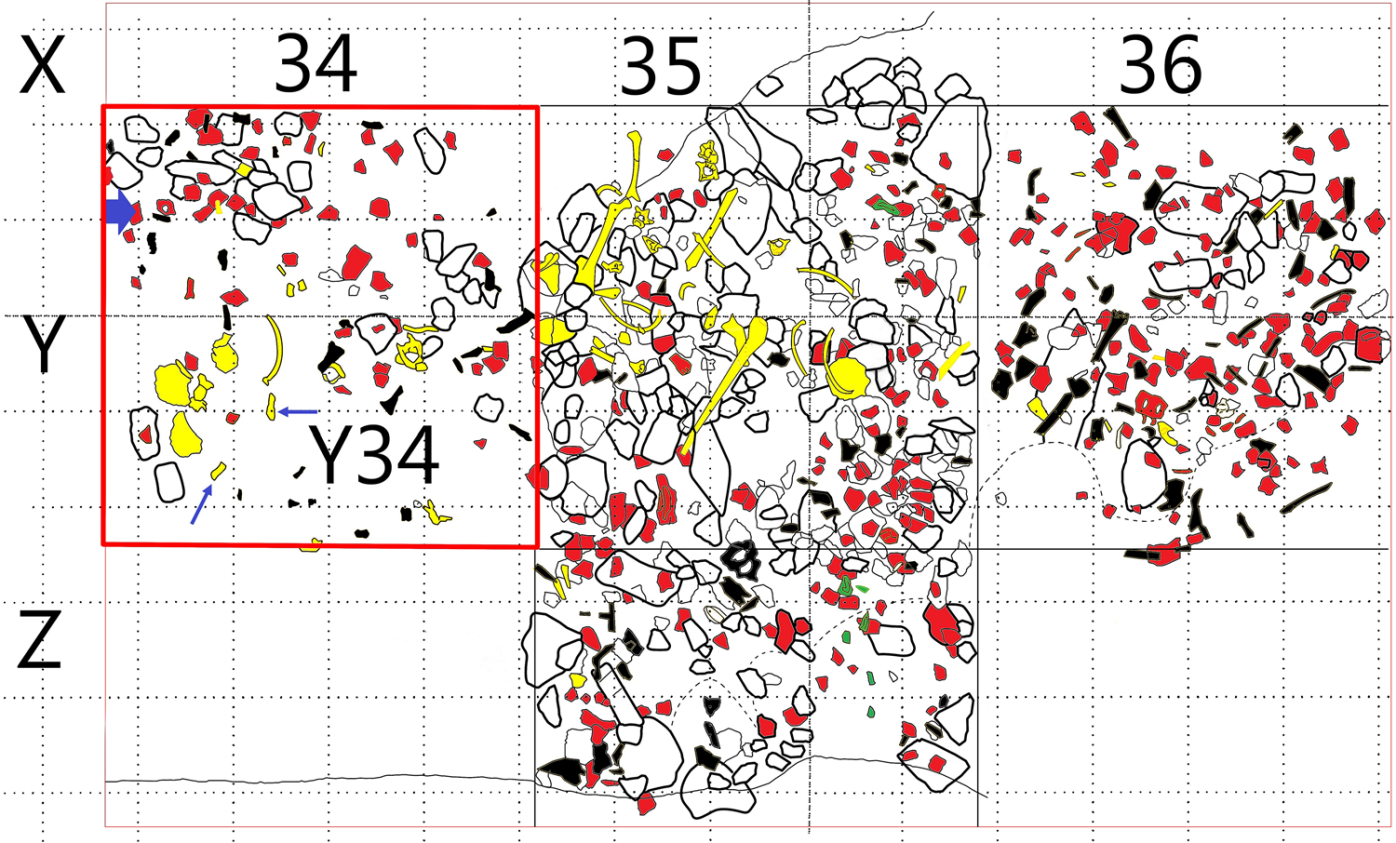
Plan of Level C4 with the location of the amber bead at square Y34 (thick blue arrow), and the location of bone samples radiocarbon dated for this paper (thin blue arrows). Squares are 1 m side. Red: pottery; yellow: bones; green: flint; black: stone. Plan edited by D. Pérez L’Huiller.

**Figure 4:**
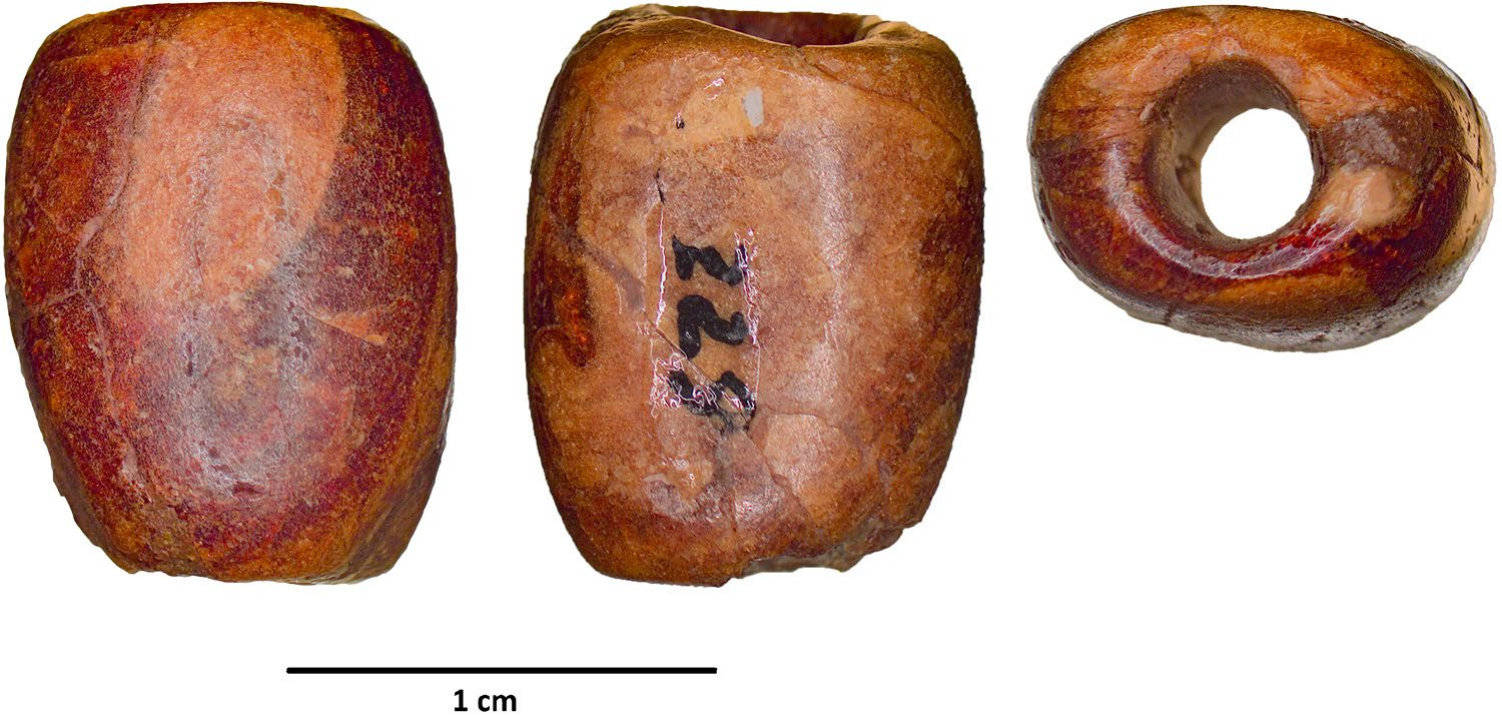
Amber bead from Cova del Frare. Photos: C. B. González edited by M. J. Vilar Welter.

Human remains (653 inventoried, 15% teeth) were widely dispersed, with the exception of four concentrations (Fig. 2). Of these, we highlight Zone 1, which yielded a concentration of long bones; Zone 3, next to the wall, with stones on top of the bones; and Zone 4, with many infant bones and the amber bead that is the subject of this study (Figs. 3, 4). Between these four areas, the few remains tended to be clustered near the walls or fallen blocks^42,43^.

The first anthropological studies estimated a minimum of 16 individuals (4 male adults, 5 female adults, 1 juvenile and 6 infants). The disproportionate representation of skeletal bones, their fragmentation and dispersion, plus the presence of anthropic incisions on two ribs and an axis vertebra, is evidence of a secondary ritual^45^. An updated review of the anthropological study lowers the number of individuals, but never to less than 12 (8 adults and 4 non-adults)^44^. It also observes a non-random fragmentation pattern, numerous perimortal fractures on fresh bone, incised lesions in the long bones of the extremities and other parts of the skeleton, such as coccygeal bones, etc., as well as carnivore bite marks^44,46^. It appears that complex rituals were carried out at Cova del Frare, including anthropic manipulation of fresh bones.

Most of the associated finds were highly fragmented. They included fauna (53% ovicaprines, 31% bovids, 13% suidae and 3% rabbits, as well as deer remains) together with pottery, stone and bone implements and ornaments^43^.

The pottery includes sherds with smooth ribs and ovoid and cylindroid vessels with superimposed nipples (identified as part of the Vérasan group), more vessels decorated with embossed lozenges, as well as some shapes and decorations characteristic of the “Sepulcros de Fosa” culture^41–43^. The finds associated with the burials therefore reflect a transition between the Middle Neolithic of the “Sepulcros de Fosa” (4200–3300 cal BC) and the Late Neolithic of Vérasan (3200–2200 cal BC)^41,45^.

The chipped stone implements include a white flake with traces of ochre, 10 local flint trapezoids, a honey-coloured flint flake from Provence, and fragments of a large “Monegros-type” flint flake from the Ebro Valley (currently being studied by C. B. González). A trapezoidal axe with a convex edge made of nephrite or amphibolite was also found^27^. Among the awls, there were two made from *Ovis/Capra* and *Bos* sp metapodiales. Ornamental grave goods included clam shell (*Glycymeris glycymeris variabilis*) beads, two variscite beads, two bone pendants with traces of ochre, and the amber bead^43^.

**Figure 5:**
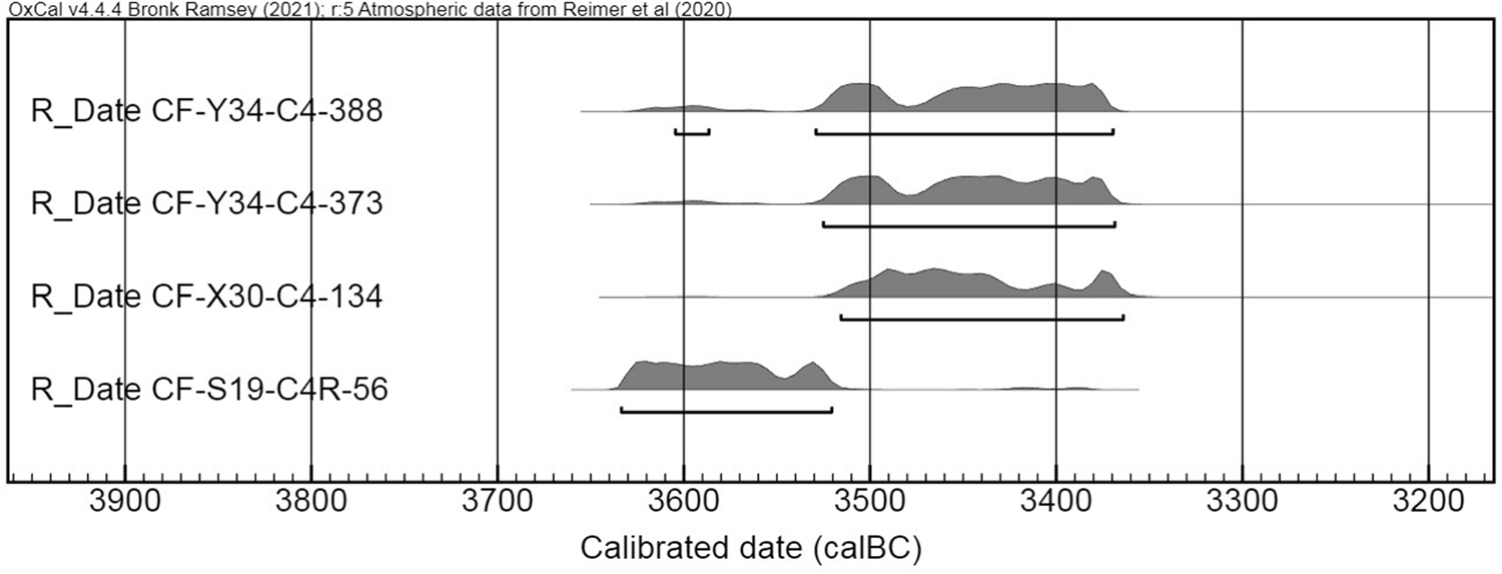
Calibrated radiocarbon dates from Cova del Frare.

The first radiocarbon date obtained from scattered pieces of charcoal found in the funerary level of Quadrant Y35 provided a date of 3480–2896 cal BC (MC-2297), confirming a chronological transition between the ‘Sepulcros de Fosa’ Culture and the Late Neolithic^41^. To narrow down the date of the particular context where the amber bead was found, two further dates were obtained on bone (CF-Y34-C4-388 and CF-Y34-C4-373) from the same quadrant (Fig. 3). Two other samples (CF-X30-C4-134 and CF-S19-C4R-56) were obtained later by J. Gibaja, M.E. Subirà and M. Fontanals in the framework of their R&D Projects (Table 1, Fig. 5). The results converge on a higher probability (95%) for the range 3527–3363 cal BC for the first three, and 3634–3521 cal BC for the fourth one.

**Table 1:** AMS Absolute dates of four samples from level C4 at Cova del Frare.

ID	Laboratory code	Sample	Radiocarbon age (BP)	Calibrate date 2σ cal BC	Sector
CF-Y34-C4-388	Beta-579371	Animal bone	4690 ± 30	(63.5%) 3475–3371 (22.2%) 3527–3485 (9.8%) 3627–3595	4
CF-Y34-C4-373	Beta-530817	Human proximal phalanx	4680 ± 30	(90.9%) 3523–3370 (4.5%) 3623–3606	4
CF-X30-C4-134	CNA 4843.1.1	Human fibula	4650 ± 30	3518–3393 cal BC (95%)	3
CF-S19-C4R-56	SUERC-97043 (GU57066)	Human left tibia	4766 ± 22	3634–3521 cal BC (95.4%)	1

Radiocarbon dates were calibrated using the internationally agreed atmospheric curve^47^, and the OxCal v4.4.4 program^48^. All dates mentioned in the text are calibrated to 2σ (95% confidence).

The analysed amber bead is barrel-shaped, with maximum dimensions of 14 × 11 mm and a longitudinal perforation of approximately 5 mm in diameter (Fig. 4).

## Results

**Figure 6:**
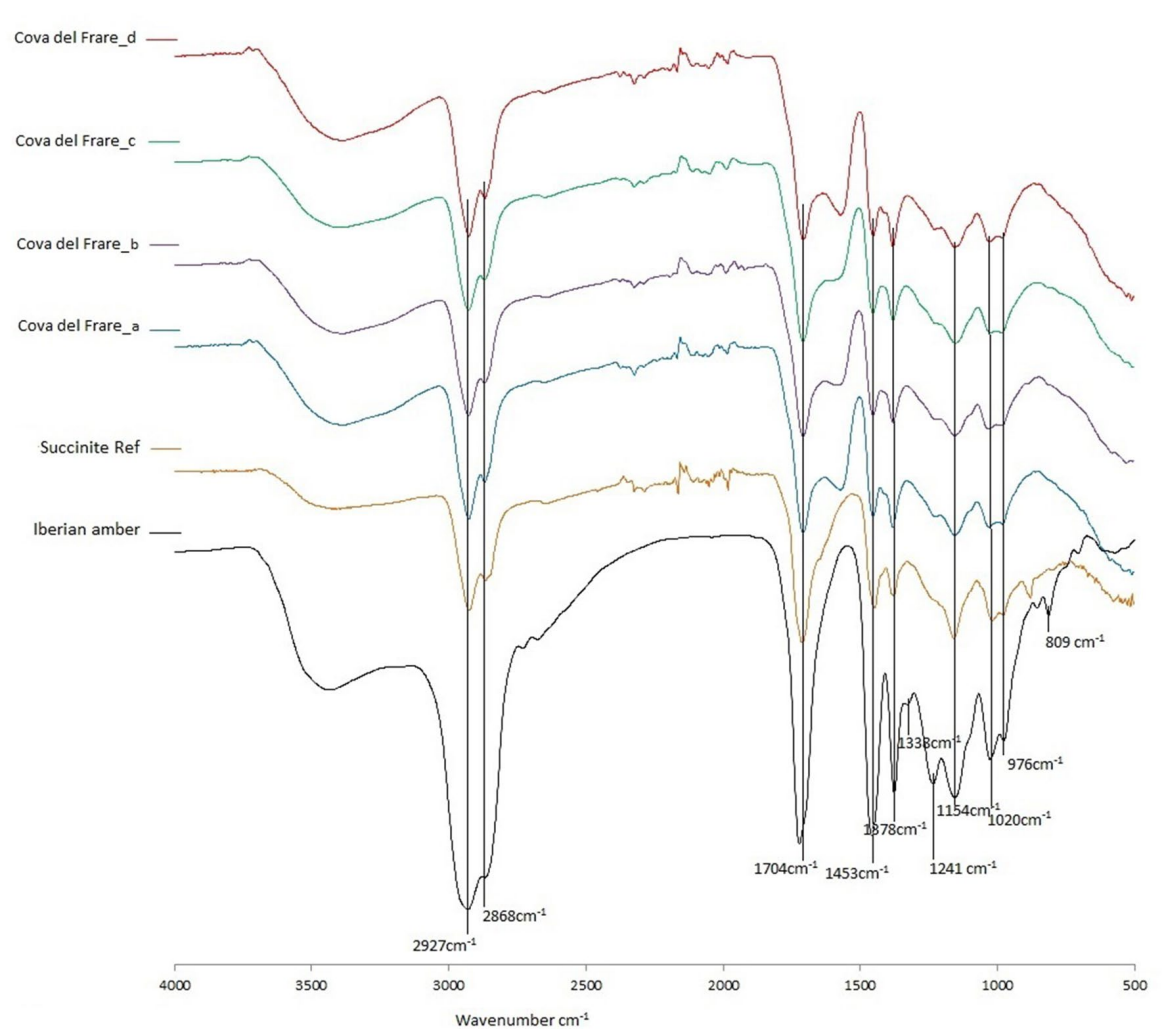
FTIR spectra of the four samples analysed from the amber bead from Cova del Frare in comparison to the reference spectra of Baltic Succinite.

The FTIR spectra obtained for the four samples are identical, showing in all cases the characteristic peaks of Class I amber, including Baltic succinite (Class Ia^49^). These resins are based on polymers and co-polymers of labdanoid diterpenes that have a regular configuration, usually including communic acid and communol, and incorporating significant amounts of succinic acid (Fig. 6).

They can clearly be interpreted using FTIR spectroscopy. In general, they present a broad band around 3400–3450 cm^−1^ due to the O–H stretching vibrations of the carboxylic acids and/or alcohols; two bands at 2924 and 2867 ± 5 cm^−1^ corresponding to the tensions of the alkyl groups, as well as the band at 1450 ± 5 cm^−1^ of the bending δ-CH2- and -CH3 and the peak at 1375 ± 5 cm^−1^, in this case due only to the bending of -CH3. The acidic carboxylic groups are reflected in the band at 1702 ± 5 cm^−1^ and in a strong band at 1157 ± 5 cm^−1^, which can be attributed to the stretching of the single C–O bond of the ester. Finally, the peaks at 1020 and 974 ± 5 cm^−1^ can be assigned to different C–O bonds. (Fig. 6; Table 2; Supplementary Material 1).

**Table 2:** Main absorbance peaks of samples analysed by FTIR.

CF_325	CF_325b	CF_325c	CF_325d	Reference Succinite_7550	
2926	2927	2926	2926	2925	ν_s_ (CH_2_) ν_s_ (CH_3_)
2867	2868	2867	2867	2866	
1704	1705	1705	1704	1710	ν_s_ (C = O)
1569		1579	1569		
1451	1451	1451	1451	1451	*δ*_*as*_(CH_3_) *δ*_*s*_(CH_2_)
1378	1377	1377	1378	1376	
1228–1198	1225–1198	1224–1199	1226–1193	1250–1180	*‘Baltic shoulder’*
1152	1150	1152	1153	1156	ν_s_ (C–O)
1027	1022	1028	1027	1026	ν_a_ (C–O)
977	981	975	977	977	

In the area of the spectrum useful for determining the origin of amber, the so-called “fingerprint” (between 1300 and 900 cm^−1^ where the bending of CH, CO, CN, CC, etc. bonds is reflected), the samples from Cova del Frare present an intense absorption peak at 1150–1154 cm^−1^ due to the stretching of the C–O single bond of the ester. This is preceded by a flat horizontal band between 1228 and 1193 cm^−1^, known as the “Baltic shoulder” since the first FTIR characterisation of Baltic amber by Beck and his team^50–52^. In contrast, instead of this characteristic horizontal band, Iberian Cretaceous amber presents an intense peak at 1240 ± 5 cm^−1^ due to the C–O symmetric stretching preceded by a secondary peak at 1335 ± 5 cm^−1^. Another characteristic feature of the Iberian amber absent in Baltic and Cova del Frare spectra is the peak at 805 ± 5 cm^−1^ preceded by a secondary one at 850 ± 5 cm^−1^ (Fig. 6).

## Discussion

The spectra of Cova del Frare bead differ significantly from the reference spectra of the Iberian cretaceous amber, while their resemblance with the Baltic succinite spectra is almost complete (Fig. 6).

The only difference is the peak at 1569 cm^−1^ in three of the samples from Cova del Frare, which appears as a shoulder in the fourth one and is absent in the succinite spectra. However, this peak, as well as the higher inclination of the “Baltic shoulder”, can be related to weathering processes. A likely explanation is the formation of carboxylic acids due to oxidation, as also detected in other archaeological samples^38^. Archaeological amber objects usually present a weathered surface layer which might modify their original FTIR spectra.

**Figure 7:**
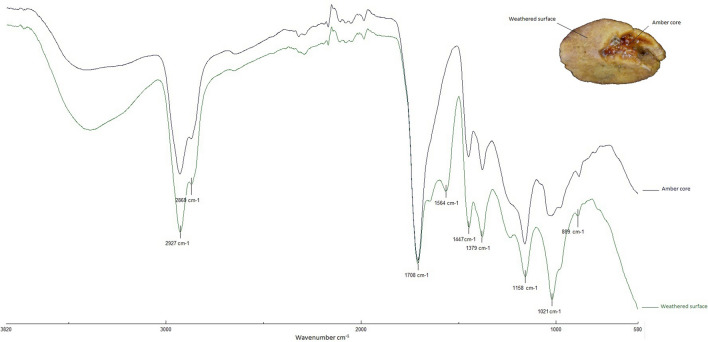
FTIR spectra of Baltic amber obtained from samples extracted from the amber core (blue) of an archaeological object and the weathered surface (green). Note that the so-called ‘Baltic shoulder’ is not significantly affected by weathering, and is distinguishable in both spectra.

In order to evaluate this surface alteration, when possible, samples are taken both from the core and the weathered surface of the artifacts. The peak at 1569 cm^−1^ observed in Cova del Frare samples has also been detected on the weathered surface of objects made of Baltic amber (Fig. 7), as well as in experimental samples heated to over 350 °C^53^. However, the “Baltic shoulder” is clearly distinguishable in both weathered and experimentally treated samples, thus remaining as a decisive proxy even in weathered archaeological samples.

Given the similarity between the four samples analysed and the characteristic Baltic succinite reference spectrum, we propose this is the origin for the material of the Cueva del Frare bead.

The archaeological region of Bòbila Madurell (Sant Quirze del Vallès)-Can Gambús (Sabadell), where the Cova del Frare site is located, is the paradigmatic reference of the coastal or Vallesià group, with pit burials and a large number and wide variety of allochthonous finds^20^. The 49 radiocarbon dates published to date confirm that both sectors belonged to a single cemetery occupied between 4100–4015 cal BC and 3655–3560 cal BC, although the Bòbila Madurell sector (4130–4010/3765–3615 cal BC) began shortly before the Can Gambús sector (4115–3980/3640–3490 cal BC)^33,34,40^.

In contrast, the group of stone slab tombs from inland Catalonia (Solsonià facies) is dated to between 4000 and 3500 cal BC with a maximum and significant concentration of probability intervals between 3800 and 3600 cal BC (26 dates)^54^ and has less allochthonous material^55^.

Studies of the available radiocarbon dates estimate a chronology for the Late Neolithic of around 3435–3110/2595–2320 cal BC^40^. They suggest a period of overlap between the first collective burials in the Late Neolithic and the last stone tombs of inland Catalonia between 3490–3215 and 3360–3120 cal BC^54^. New dates show a beginning of the Late Neolithic from 3600 to 3500 cal BC^44^.

The Baltic amber bead presented in this paper comes from a context dated to the Late Neolithic (3634–3370 cal BC). Although the use of amber in early contexts on the Iberian Peninsula is not unusual, the fact that it originated in northern Europe is completely exceptional.

In the Iberian Peninsula there are more than 160 locations with palaeontological amber outcrops^56,57^ (Fig. 1). Most of these amber deposits are Albian in age (Early Cretaceous) and only a few localities in Asturias and Catalonia date to the Late Cretaceous. Likewise, only two localities with amber from the Late Triassic Period are known, both in Alicante^56^.

In general, the amber deposits are distributed in a strip that runs from the east to the north of the Iberian Peninsula, broadly corresponding to the coastline during the Early Cretaceous. Specifically, at a distance of about 50 km from the Cova del Frare there are various amber deposits, initially thought to be fossil amber from the conglomerates of the Sant Llorenç del Munt mountain (Fig. 1)^56^.

The amber outcrops in Catalonia are found in the Maastrichtian (into the Garumnian facies) associated with lignite deposits that have been exploited until recent times in the Pre-Pyrenees (mainly in the Figols-Vilada area and at Isona in the Tremp Basin)^58^. Amber usually appears in small amounts as tiny drops in the greyish muddy layers and occasionally as masses up to several centimetres. No bioinclusions have been found in the samples recovered^56,58^.

The main amber deposits—larger than those of Catalonia—are documented in the Central Asturian Depression (CAD), the Basque-Cantabrian Basin (BC) and the Maestrat Basin (MB), with well-known palaeontological amber deposits such as those of El Soplao or San Just, where specimens weighing about 1 kg have been found^59,60^. These Iberian deposits were exploited by prehistoric communities from the Upper Palaeolithic onwards. The earliest archaeological pieces analysed are the Aurignacian, Gravettian and Magdalenian fragments found in caves, some of which have evident anthropogenic marks^38,61,62^. These local resources continued to be used during the second half of the fourth and the first half of the third millennia BC in megalithic monuments in which Bell Beaker finds were also documented^61,63^. The most recent context in which the exploitation of local amber has been documented is the megalithic complex of Los Lagos I (Cantabria), where the remains of amber were found in the chamber. This assemblage provided a date of 1731–1404 cal BC^64^ (Fig. 1).

Baltic amber is currently believed to have arrived at the Iberian Peninsula much later. In the megalithic monument of Larrarte (Guipúzkoa), an amber bead was documented among predominantly Chalcolithic grave goods, with abundant stone tools, sherds of Bell Beaker and plain pottery, and discoidal lignite and stone beads^65^. Unfortunately, none of the twelve buried individuals could be dated, due to a lack of collagen, and the only dates for the dolmen analysed by the Teledyne Isotopes laboratories (5360–4052 cal BC and 4241–3539 cal BC) come from two pieces of charcoal collected outside the chamber, in the mound, with the inherent problems of “old wood” and the uncertainty derived from the lack of contextual association^65^. A safer instance is the context of the Baltic amber bead found in Burial 38 at La Almoloya (Murcia), a double burial in *pithos* of exceptional opulence^66^. The male individual, 35–40 years old, is associated with a small amber bead of Baltic origin, not only the sole evidence of amber in the entire Argaric area, but also the first evidence of the arrival of Baltic amber with a definite context. The individual provided a date of 1738–1534 cal BC^66^.

With the exception of these two pieces, the amber beads of Baltic origin are concentrated in the northeast of the Iberian Peninsula^9^. In this area there are seven archaeological sites with analysed pieces of Baltic origin (Fig. 1). These are the beads from Cabana del Moro de Colomera, Pedra Cabana, El Bosc, La Pera, El Garrofet, Muricecs, and Fosa del Gegant. However, most of these contexts are collective burials excavated in the early twentieth century for which we do not have detailed stratigraphic or contextual information, and it is not possible to associate pieces with particular individuals. However, given the associated archaeological finds (ranging from Bell Beaker items to bronze objects with between 10 and 12% Sn and even iron or vitreous paste beads)^39^, all these cases can be considered as later than the Cova del Frare.

Against this background, the Late Neolithic (3634–3363 cal BC) collective burial phase of the Cova del Frare provides the earliest evidence of Baltic amber in Western Europe. In the case of the northeastern Peninsula, Baltic amber use lasted throughout the Chalcolithic and Bronze Age without penetrating interior or southern Iberia until later, during the Late Bronze/First Iron Age (with the exception of the aforementioned La Almoloya bead dated to the middle of the 2nd millennium BC). Even though just a single bead, this finding places the occurrence of Baltic amber in Western Europe more than a millennium earlier than previously thought. Crucially, it also compels us to question the traditionally assumed link between Baltic amber and the spread of Bell Beaker culture during the 3rd Millennium BC^67^.

In northern Europe, where Baltic amber occurs naturally, this raw material had been used since the Palaeolithic and Mesolithic periods, although it was during the 4th and up to the 3rd millennium BC that its exploitation increased considerably. At that time amber was distributed throughout the area in which the Funnel Beaker Culture (FBC) developed, spanning present-day Denmark, the Netherlands and the north of present-day Germany and Poland. It was used to make buttons with V-shaped perforations, discs, beads, pendants and some figures or beads shaped like battle axes, demonstrating the importance of the axe as symbol^67^.

Amber was worked in large quantities from the Neolithic on (with more than 50,000 Neolithic amber beads documented)^37,68^. Thus, large deposits with amber objects have been found, such as that documented at Sortekærs Mose, Læsten or Mollerup in Denmark. Some of these deposits came to contain more than 8 kg of amber, evidencing the intense exploitation of this local raw material, which was easily accessible on the coast in the Neolithic period.

During the 3rd millennium BC, some Baltic amber beads began to appear in other European regions. Du Gardin^37^ began to document pieces of amber from between 3000 and 2600 cal BC at ten French sites (Villevenard, Oyes, Charavines, Annecy, Epone, Flavacourt, Méréaucourt, Mériel, Chouilly and Ay Champagne); of those analysed, one bead from Oyes, another from Charavines and five from Layers 4 and 5 at Méréaucourt were found to be Baltic succinite^37^. Before this paper, these constituted the earliest evidence of Baltic amber in Western Europe, despite France having local amber resources^37,69^. Nevertheless, there is evidence of the exploitation of local resources in earlier and later times. Like in Iberia, the earliest amber ornaments in France date to the Palaeolithic. At Isturitz, 27 pieces of amber with working marks have been documented, including two pierced earrings^70^ made with local resources^71^. The earliest amber objects documented so far in Italy date back to the end of the 4th millennium BC^12^ although the use of local amber (simetite) is proposed for these early objects. The earliest evidence of the arrival of Baltic amber to Italy dates to 1800 BC^13,72^.

Between 2600 and 2200 cal BC, there are 21 French sites were amber is found^37^. The analysed beads—two from Thiré, two from Narbonne, one from Xanton-Chassenon and another from Montagnac-Montpezat—revealed a Baltic origin^37^, while two from Narbonne remain unidentified. Unfortunately Du Gardin does not publish the spectra analysed, so we cannot compare them with other sources of amber. At this time amber also appears at the archaeological sites in the south of France closest to the Iberian Peninsula, such as Salses, Saint-Pargoire, Saint Maurice-de-Navacelles, Châteaurenard, Montpezat or Narbonne (Fig. 1). In fact, this area has the greatest concentration of amber pieces in France, with another small group in Brittany, in the Parisian basin, following the course of the River Seine, or in the Lyon area, following the course of the River Rhône. It is also significant that, as in the Iberian Peninsula, the use of amber decreased considerably with the dawn of the Bronze Age. Between 2200 and 1800 cal BC, amber has only been documented at five French archaeological sites, although this increases to 19 in a second phase between 1800 and 1400 cal BC^37^.

We can relate this situation to the close links between the northeast of the Iberian Peninsula and the south of France beginning in the Neolithic^27,73–75^. Evidence for these includes the presence of Barremian-Bedoulian flint from the Vaucluse region in northeastern Iberia, as well as other exogenous materials, such as Sardinian obsidian and Alpine rocks^24,27,76^. These connections, which become especially evident with the Vérasan group and other Late Neolithic–Chalcolithic groups who shared similar material culture, continued until at least the late 3rd millennium BC^77^. The Pyrenees mountains are currently seen as a barrier constituting the dividing line between two countries. During prehistory, however, they were far from being perceived as a border, but rather as a zone of contact and interaction for social groups from at least the Early Neolithic. This became very evident in the Middle Neolithic and especially in the Late Neolithic and Chalcolithic Vérasan, a group that twinned the populations on both sides of the range^44^.

Recent studies suggest that not all the finds in the Iberian northeast came from the same trading network, but rather from different networks resulting from contacts with various communities with a relatively distinct temporal validity^22,24,33^. In any case, the amount of exogenous materials involved in these networks in which amber seem to have participated only occasionally, would have begun to decrease from 3655 to 3550 cal BC, suggesting the beginning of what may have been a sudden collapse of the entire network. These dates appear to coincide with profound changes in funerary practices in terms of structures, burial methods and grave goods. From that moment on, collective burials in different places—especially dolmens, hypogea and caves—succeeded previous practices^34,40^.

## Conclusions

In this article we presented an amber bead found in a funerary context in Cova del Frare (Barcelona) and radiocarbon dated to the Late Neolithic (3634–3363 cal BC). FTIR analyses and comparison to reference materials demonstrated that the amber is Baltic, and therefore was transported from northern Europe via the trade networks of the “Sepulcros de Fosa” culture, shortly before its presumed collapse^34,40^.

These results place the arrival of Baltic amber on the Iberian Peninsula more than a millennium earlier than previously established, and constitute the earliest evidence for this material in Western Europe. The broader context is one of close contacts between the communities of on both sides of the Pyrenees, as evidenced from the Neolithic until at least until the end of the 3rd millennium BC.

At the same time, it is interesting that the bead dates to a period where the abundance of exogenous materials begins to decrease. This makes it plausible that even earlier Baltic amber may be awaiting discovery in Western Europe.

## Methods

Four small samples (< 0.1 g) were taken for Fourier Transform Infrared Spectroscopy (FTIR) characterisation. This was performed at the University of Granada’s Centre for Scientific Instrumentation using a Jasco 6200 FTIR spectrometer coupled with an attenuated total reflectance system (ATR), making pellet preparation unnecessary. The samples were analysed 50 times in the 4000–400 cm^−1^ range with a resolution of 4 cm^−1^. The spectra, which are presented in infrared transmission, were processed with the Spectra Manager v2 software.

### Supplementary Information

Supplementary Information.

## Data Availability

All data generated or analysed during this study are included in this published article (and its supplementary information files).
